# Levels of Urinary Biomarkers of Oxidatively Generated Damage to DNA and RNA in Different Groups of Workers Compared to General Population

**DOI:** 10.3390/ijerph16162995

**Published:** 2019-08-20

**Authors:** Giovanna Tranfo, Enrico Paci, Mariella Carrieri, Enrico Marchetti, Renata Sisto, Monica Gherardi, Francesca Costabile, Lisa Bauleo, Carla Ancona, Daniela Pigini

**Affiliations:** 1 Department of Occupational Medicine, Epidemiology, Occupational and Environmental Hygiene, INAIL Research, via Fontana Candida 1, Monte Porzio Catone, 00078 Rome, Italy; 2 Department of Cardiac, Thoracic, Vascular Sciences and Public Health, University of Padova, via Giustiniani 2, 35128 Padova, Italy; 3 CNR-ISAC—Italian National Research Council, Institute of Atmospheric Science and Climate, via Fosso del Cavaliere 100, 00133 Rome, Italy; 4 Department of Epidemiology, Lazio Regional Health Service, Via Cristoforo Colombo 112, 00147 Rome, Italy

**Keywords:** DNA and RNA oxidation, HPLC-MS/MS, biological monitoring, effect biomarkers, occupational exposure

## Abstract

(1) *Background*: The products of guanine oxidation in DNA and RNA excreted in urine are 8-oxo-7,8-dihydroguanine (8-oxoGua), 8-oxo-7,8-dihydroguanosine (8-oxoGuo), and 8-oxo-7,8-dihydro-2’-deoxyguanosine (8-oxodGuo). Despite intra and inter-individual variability, it is possible to identify situations that significantly increase the levels of these compounds when comparing urinary concentrations of some workers to those of the general population. (2) *Methods*: urines from gasoline pump attendants (58 from Saudi Arabia and 102 from Italy), 24 workers of a fiberglass reinforced plastics plant, 17 painters and 6 divers were analyzed by HPLC/MS-MS. To test the individual variability, two subjects provided daily samples for one month, and 132 urine samples from the general population were analyzed. (3) *Results*: We summarized the results for each biomarker, and found the following were statistically higher than in the general population: 8-oxoGua in fiberglass and Italian gasoline workers; 8-oxodGuo in fiberglass and both Saudi Arabian and Italian gasoline workers; 8-oxoGuo in fiberglass workers, both Saudi Arabian and Italian gasoline workers, and painters after the working shift. (4) *Conclusions*: these results confirm that both 8-oxodGuo and 8-oxoGuo are valuable biomarkers for occupational exposures to dangerous chemicals and seem to suggest that 8-oxoGuo, related to RNA oxidation, is a suitable biomarker to evaluate short term, reversible effects of occupational exposures even within the health-based limit values.

## 1. Introduction

Oxidatively generated damage to DNA and RNA plays an important role in cancer development, cardiovascular and neurodegenerative diseases, diabetes, pulmonary fibrosis, and more [1,2,3,4]. Oxidative modifications of DNA nucleobases are generated by reactive oxygen species (ROS); ROS can be produced by several endogenous and exogenous sources, including metabolic processes, air pollution, exposure to solar radiation and ionizing radiation, lifestyle, including smoking, alcohol consumption and poor diet, drugs, and some occupations [5].

On DNA, guanine is the most susceptible to oxidation, because it has a low redox potential, leading to the formation of 8-oxo-7,8-dihydroguanine (8-oxoGua), the most common lesion. 8-oxoGua is generated by radical reactions with ^•^OH, one-electron oxidants and vicinal pyrimidine peroxyl radical, and by reaction of ^1^O_2_ with guanine giving rise to 8-oxoGua, in cellular DNA [6]. 8-Oxo-7,8-dihydro-2′-deoxyguanosine (8-oxodGuo) is an important DNA lesion product that can be generated by hydroxyl radicals, singlet oxygen, and one-electron oxidants [7]. Once formed, it is essential that 8-oxoGua does not remain in the genome. The multiple repair pathways that undertake this task are base excision repair (BER) and nucleotide excision repair (NER) [8]. 8-oxoGua and 8-oxodGuo found in human urine originates from DNA repair mechanisms and possibly also from turnover of oxidatively damaged DNA [9].

Oxidative modifications of RNA guanine can lead to the formation of both 8-oxo-7,8-dihydroguanine (8-oxoGua) and of 8-oxo-7,8-dihydroguanosine (8-oxoGuo). RNA is single-stranded and its bases are therefore less protected by hydrogen bonding and more easily accessible to ROS than DNA bases. RNA has relatively less association with proteins and has an extensive sub-cellular distribution and cytoplasmic RNA is in close proximity to mitochondria, where the majority of ROS is generated. Oxidative modifications to mRNA results in disruption of translation and hinders protein synthesis, which can cause cell deterioration or even cell death [10]. Products of RNA damage have not received much attention, [11] presumably because of the assumption that damaged molecules do not accumulate due to the transient nature of RNA. However, damage by ROS occurs in minutes, while most human mRNA persists much longer (average half-life 10 h). Moreover, stable RNA species (mainly ribosomal RNA and transfer RNA) constitute the majority of cellular RNA and are not degraded during exponential growth [12].

Attacks of ROS on DNA and RNA lead to the urinary elimination of 8-oxoGua, 8-oxodGuo, and 8-oxoGuo, which are considered biomarkers of oxidatively generated damage on DNA and RNA: they are always detectable in the general population as the results of the exposure to oxidative stress agents, that can have different origins. In this paper we used the nomenclature suggested in [6].

In urban environments the general population is exposed to particles generated from traffic (PM_2.5_) and from domestic heating, the latter occurring in the winter [13]. Particulate matter from traffic exhausts contains polycyclic aromatic hydrocarbons (PAHs) and can therefore produce a significant amount of ROS increasing oxidatively generated DNA and RNA damage [14,15]. Residential exposure to pollutants released in the proximity of an industrial plant, can also contribute to oxidative stress [16,17].

Different studies indicate that exposure to hyperbaric oxygen (HBO) causes oxidatively generated damage to DNA, which is rapidly repaired by activating an adaptive protection against this additional oxidative stress. Repeated exposure would modify the degree of damage [18] and cause an increased production of free radicals [19].

Oxidative stress markers have also been studied in the context of occupational exposure to chemicals such as aluminum [20], wood smoke inhaled by firefighters [21], pesticides in farmers [22,23], and metal oxide nanoparticles [24].

In this study, the oxidized nucleobases and nucleosides excreted in urine, namely 8-oxoGua, 8-oxodGuo and 8-oxoGuo, measured by HPLC-MS/MS, were compared in different groups of subjects occupationally exposed to chemicals and to those of a group of general population volunteers in order to understand which of the three markers is best suited for a given occupational exposure setting, which of these markers is most sensitive to short-term exposure variation and to inform us about the relative importance of DNA versus RNA damage.

## 2. Materials and Methods

### 2.1. Study Population

Oxidatively generated damage to nucleic acid was studied in seven different groups of subjects, three of which were divided into subgroups.

Group 1 consisted of 29 gasoline pump attendants from Saudi Arabia, who performed extended work shifts (10–12 h) under temperatures above 40 °C and were therefore exposed to an increased risk of inhalation of gasoline vapors. Smokers were not allowed to smoke during the work shift. The urine samples were collected at the end of the working shift in the period of July–August 2014 [25].

Group 2 consisted of 102 gasoline pump attendants from Italy, who performed an 8 h work shift, exposed to low benzene levels (range < 1.5–80 µg/m^3^, mean 15 µg/m^3^, media 7 µg/m^3^), and were monitored at the end of the work shift in the period May 2012–May 2017 [25].

Group 3 consisted of 24 workers of a fiberglass reinforced plastics manufacturing plant who were exposed to styrene vapors with a mean concentration above the TLV—TWA of 85 mg/m^3^ (IARC classification is 2A, probably carcinogenic to humans) [26]. Workers wore disposable respirators for protection against dust and fibers. The urine samples were collected after working in the period 2011–2013 [27].

Group 4 consisted of 17 ship painters from Bangladesh working in Italy, exposed to organic solvents (toluene, xylene, etc.) and to other substances such as diluents and additives (eptan-2-one, 2-butossiethyl acetate, 1-methyl-2metossiethylacetate, butanone, ethyl acetate, n-butyl acetate), with potential exposure to solvent values almost equal to the mixture relative exposure limit. The urine samples were collected before and after the work-shift in June 2018. Workers wore full facepiece respirators for protection against dust and organic vapors during the working shift.

Group 5 consisted of 6 subjects exposed to an hyperbaric atmosphere, who performed an immersion in the Bracciano lake, near Rome, during a controlled experiment. They wore a wetsuit, compensation jacket, tank, regulators and a dive computer Galileo SOL. The dive lasted for 30 min at 20 m deep. The tank had a volume of 15 L, filled with external air at a pressure of 200 atm. Divers used up to 100 atm of the given air. The temperature of the water was 12 °C at the bottom, 32 °C at the surface. Multiple urine samples were collected before diving and until 12 h after diving, in June 2018.

Group 6 consisted of two researchers walking in the open air in the center of Rome for about 6 h/day carrying a backpack containing air quality measuring instruments. Daily samples were provided over the course of one month, to test the intra-individual variability. The subjects participated in the larger “Carbonaceous Aerosol in Rome and Environs (CARE)” experiment, which had the objective to evaluate the health impact of fine and ultrafine particles with different methods. The urine samples were collected from 27 January to 28 February 2017 [17] and complete results for the nucleic acid oxidation biomarkers are reported here.

Group 7 consisted of a randomly selected general population sample of 132 subjects who had been living for at least 10 years in the same area of Central Italy; first morning urine samples were collected between May 2013 and December 2014, in the framework of a larger Human Biomonitoring study called ABC and the nucleic acid oxidation study was carried out later and published in 2017 [28]. For each subject information related to age, occupational history, and living and smoking habits were collected by questionnaire. Before providing the urine sample all subjects gave written informed consent to participate in the study. The study was conducted in accordance with the Declaration of Helsinki, and the protocol was approved by the Local Ethics Committee of AUSL RM /E (registered as n.14/13 of 16 April 2013).

### 2.2. Urine Sample Collection

Collection and processing of urine samples always followed the same standardized procedure: samples were collected in sterile plastic containers and transported refrigerated to the laboratory within 12 h. Once in the laboratory, samples were divided into at least three aliquots, that were stored in polypropylene tubes at −20 °C until analysis, to avoid thawing and refreezing of the same sample to perform the determination of different analytes. About the stability of the analytes during storage, Poulsen et al. reported stability of 8-oxodGuo at −20 °C for 6 y [29] while Loft et al. for 15 y [30]. Samples were thawed in lukewarm water, at about 37 °C.

### 2.3. Chemicals and Supplies

The analytical reference standards of 8-oxoGua. 8-oxodGuo and 8-oxoGuo were purchased by Spectra 2000 s.r.l (Rome, Italy). The isotope labeled internal standards (^13^C^15^N_2_) 8-oxodGuo and (^13^C^15^N_2_) 8-oxoGuo were obtained from CDN Isotopes Inc. (Pointe-Claire, QC, Canada). (^13^C^15^N) 8-oxoGua (98%) was obtained from Cambridge Isotope Laboratories Inc. (Tewksbury, MA, USA).

Glacial acetic acid 30% NH_3_. Dimethyl sulfoxide. Sodium hydroxide solution (50%–52% in water) and CHROMASOLV^®^ gradient grade ≥99.9% Methanol and Acetonitrile for HPLC/MS ≥99.9% carbon disulfide low benzene content were obtained from Sigma Aldrich (Saint Louis, MO, USA). Purified water was obtained from a Milli-Q Plus system (Millipore Milford, MA, USA). Anotop 10LC syringe filter device (0.2 µm pore size, 10 mm diameter) were purchased from Whatman Inc. (Maidstone, UK). A Kinetex Polar C18 column 100 A. (150 × 4.6 mm, 2.6 µm) supplied by Phenomenex (Torrance, CA, USA) were used throughout the study.

The urine samples were analyzed on a Series 200 LC quaternary pump (PerkinElmer, Norwalk, CT, USA) coupled with an AB/Sciex API 4000 triple-quadrupole mass spectrometry detector equipped with a Turbo Ion Spray (TIS) probe.

### 2.4. Analytical Methods

The concentration of 8-oxoGua, 8-oxoGuo, and 8-oxodGuo was determined by isotopic dilution HPLC-MS/MS following the method described by Andreoli et al. 2010 with some modifications [31]. The urine sample were centrifuged and analyzed. The precursor→product ionic transitions monitored (positive ion mode) were 168.0→140.0 and 171.0→143.0 for 8-oxoGua and its internal standard ((^13^C^15^N_2_) 8-oxoGua) 284.3→168.0 and 287.13→171.1 for 8-oxodGuo and its internal standard ((^13^C^15^N_2_) 8-oxodGuo), 300.24→168.2 for 8-oxoGuo and 303.24→171.0 for the internal standard ((^13^C^15^N_2_) 8-oxoGuo), respectively. The 1.5 version of Analyst^®^ software (AB Sciex, Framingham, MA, USA) was employed for instrument control.

The final concentration of the analytes was expressed in µg/g of creatinine to normalize values with respect to urine dilution variability. Urinary creatinine was determined by the method of Jaffè using alkaline picrate test with UV/Vis detection at 490 nm [32].

### 2.5. Statistical Analysis

All the statistical analyses were performed using the statistical software SPSS (version 25, IBM, Armonk, NY, USA) and R (version 3.5.3 (2019-03-11), R Foundation for Statistical Computing, Vienna, Austria).

The urinary concentrations of 8-oxoGua, 8-oxodGuo, and 8-oxoGuo were treated as continuous numerical variables. The normality of their distribution was tested using the Kolmogorov-Smirnov normality test. In order to evaluate the statistical significance of the differences between the different groups an ANOVA test was performed. Groups with a poorer sample size (group 5 and group 6, *n* = 6 and *n* = 2 respectively) were excluded from the ANOVA.

For each biomarker an ANOVA test was performed, in which the variables smoking, sex, and ethnicity were considered as factors, whilst the age was kept into account as a covariate. For paired comparison among the groups, a post-hoc Tukey test was applied, and the difference between the means was considered significant if ≤0.05. At the aim of quantitatively determining the average difference in biomarker concentrations, a linear regression model was applied, in which the log-transformed biomarker concentration was the outcome and the group was the explanatory variable.

To evaluate the sensitivity of the three biomarkers to the short-term effect of the exposure, a paired comparison (paired *t*-test) was applied between the samples collected before and after the exposure, for groups 4 (ship painters) and 5 (divers).

## 3. Results

The characteristics of the six groups studied are reported in Table 1.

The inter and intra individual variability is reported in Table 2, separately for males and females. We considered a measure of the intra individual variability the coefficient of variation (% CV) of the three biomarkers in the multiple samples of two subjects in group 6, while the same parameters in the general population (68 men and 64 women) are considered a measure of the inter individual variability.

The data show that the inter- and intra-individual variabilities are of the same order of magnitude and that intra individual can be more than 100% in some cases. Andreoli et al. [33] found a comparable or even higher inter-individual variability for 8-oxoGuo (GSD/GM 75%) and 8-oxodGuo (GSD/GM 104%).

Table 3 shows the descriptive statistics of the urinary oxidation biomarker concentrations expressed in µg/g creatinine in all the six groups considered. Values are presented as mean with the standard deviation, 5th, 50th (median value), and 95th percentiles.

The normality of distribution, tested using the Kolmogorov-Smirnov test gave a statistically significant result for each of the three variables (*p* < 2.2 × 10^−16^).

The results of the ANOVA are reported in Table 4.

The results of the linear regression model are reported in Table 5. The regression coefficients represents the average increase with respect to the average concentration of the group 7, representing the baseline.

It can be noticed that only in the case of the 8-oxoGuo biomarker, the concentration always increases in the different group of exposed subjects with respect to the control group.

Smoking was a significant factor only in the case of the 8oxoGua biomarker. For the other two biomarkers, as the smokers were present approximately in the same percentage in each group, in comparing the groups this factor gave no significant effect. Moreover, this finding confirms previous results [28]. A significant effect of age was observed in the case of the 8oxoGuo, more precisely, an increasing concentration is associated with a higher age. The best fitted coefficient of the 8oxoGuo with age was 0.14 (standard error 0.05, upper limit CI 95% = 0.24, lower limit CI 95% = 0.039, *p* = 0.007).

The linear trend of the data with age has been subtracted from the 8oxo-Guo concentration, in other words the term

Β × (age − age_0) with age_0 = 20 years(1)

was subtracted from the measured concentration.

**Figure 1:**
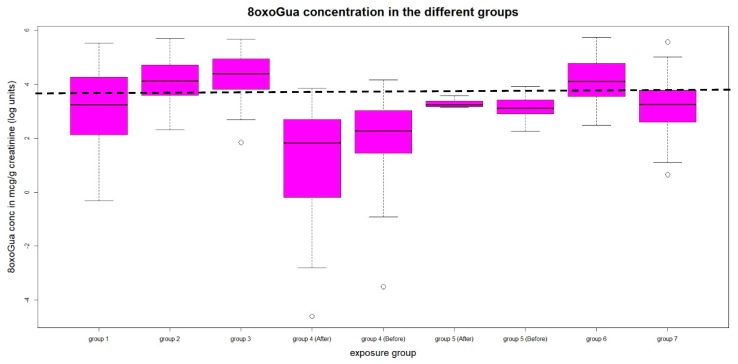
Urinary concentrations of 8-oxoGua.

**Figure 2:**
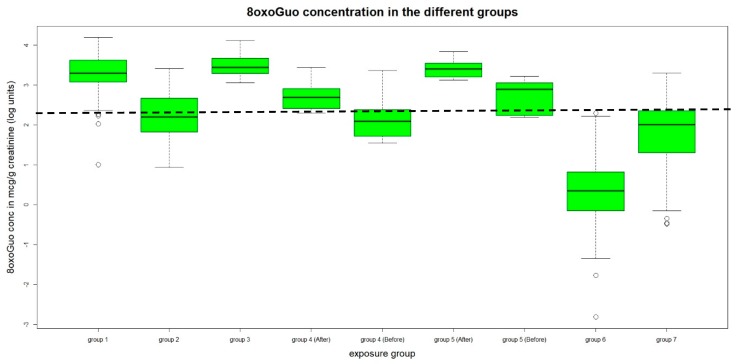
Urinary concentrations of 8-oxoGuo.

**Figure 3:**
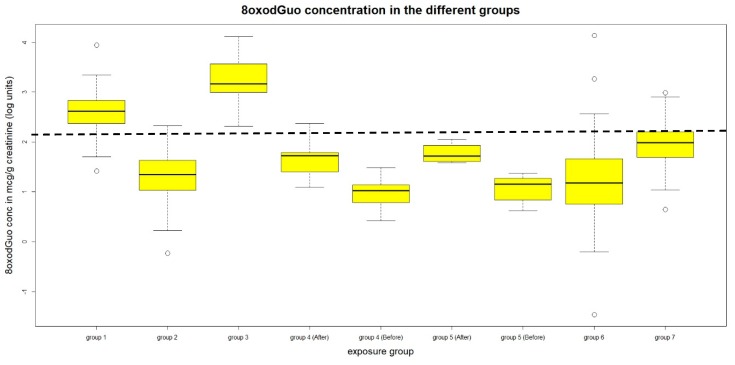
Urinary concentrations of 8-oxodGuo.

The concentration distribution of the three biomarkers in the different groups are shown in the box plot of Figure 1, Figure 2 and Figure 3 respectively for 8-oxoGua, 8-oxoGuo and 8-oxodGuo. The concentrations of the 8-oxoGuo are de-trended with respect to the age variable as previously described. The dotted straight line represents the 75th percentile of the urinary concentration of the considered biomarker in the general population (group 7), to highlight the groups whose levels exceed it.

A post-hoc Tukey test was applied in order to compare the different group of exposed subjects to the general population.

Group 1, Gasoline pump attendants from Saudi Arabia: the urinary concentrations of both 8-oxodGuo and 8-oxoGuo are significantly higher than those of group 7.

Group 2, Gasoline pump attendants from Italy: the concentration of 8-oxoGua, 8-oxo Guo (corrected for age), and 8-oxodGuo are higher than that of group 7.

Between the above groups, the differences of the urinary concentrations of 8-oxoGuo and 8-oxodGuo are significantly higher in Saudi Arabian than in Italian workers.

Group 3, Fiberglass workers: for all the three measured biomarkers, urinary concentrations are significantly higher than those of group 7.

Group 4, Ship painters: the urinary concentrations of 8-oxoGuo after the working shift is significantly higher from that of group 7.

Very interestingly a significant increase in the biomarker concentrations was observed by comparing the same subjects before and after the exposure to diving (group 5) and the exposure to painting (group 4). More precisely, the paired *t*-test was significant for 8-oxoGuo (*p* = 0.0024) and for 8-oxodGuo (*p* = 0.0002) in the case of divers and for 8-oxoGuo (*p* = 1.8 × 10^−5^) and for 8-oxodGuo (*p* = 6.6 × 10^−6^) in the case of painters.

## 4. Discussion

The three urinary biomarkers determined in this study show a significant intra and inter-individual variability. This variability is clearly due to the many parameters that influence the oxidative challenge to the single individuals, like genetic factors, enzyme induction and environmental exposures. Given two persons having the same external exposure and the same DNA damage, the one with the/more efficient repair system would have higher levels of biomarkers in urine. So, while on a group level these biomarkers could serve as markers of exposure, on an individual level they could be interpreted as markers of susceptibility (with high levels a “good” sign). On the other side we know that these biomarkers do not follow any circadian rhythm [31].

Despite this variability, results show that it is possible to identify situations that produce more (or less) oxidative stress through the statistical comparison of the mean levels of different group of subjects, both for 8-oxoGuo and 8-oxodGuo. For 8-oxoGua we apparently do not have enough information in order to give an interpretation for the values measured in the different groups.

The gasoline pump attendants, having the urinary concentrations of both 8-oxodGuo and 8-oxoGuo higher than those of the general population, were exposed to benzene: the exposure had been assessed by biological monitoring through the determination of the urinary metabolite of benzene S-phenyl-mercapturic acid (SPMA) for the Saudi Arabian workers and through both environmental and biological monitoring for the Italian workers [25].

The fiberglass workers showed statistically significant higher values than group 7 for all the three biomarkers: they had been exposed to VOCs, acetone, fiberglass, and overall, styrene. According to the harmonized classification and labelling approved by the European Union, this last substance causes damage to organs through prolonged or repeated exposure, is a flammable liquid and vapor, causes serious eye irritation, is harmful if inhaled, is suspected of damaging the unborn child and causes skin irritation; besides its IARC classification is 2A (Probably carcinogenic to humans) [26].

The painters, having the mean 8-oxoGuo value after the work shift higher than the general population group, were exposed to a complex mixture of volatile organic solvents plus additives and diluents resulting in the painting activity being recognized as a Class 1 carcinogen by IARC [26]. Biomonitoring was carried out measuring unchanged solvents and benzene, toluene and xylene metabolites in the urine. Results increased after the working shift evidencing incomplete protection, and possibly skin absorption (unpublished data).

The divers were exposed to an increased pressure and breathed a concentration of oxygen about three times higher than the normal. A maximum value was found after 4 h for the three studied biomarkers for all subjects (unpublished data). The rise of the biomarkers 8-oxoGuo and 8-oxodGuo concentrations after the diving session detected by the t-test confirms the presence of an effect. This finding supports the hypothesis that the 8-oxoGuo and the 8-oxodGuo can be used as early biomarkers of effect in subjects exposed to oxidative stress factors.

If we summarize the results for each variable, the groups that have statistically higher values than the general population are:

For 8-oxoGua, fiberglass workers and Italian gasoline workers (two groups);

For 8-oxodGuo, fiberglass workers and Saudi Arabian and Italian gasoline workers (three groups);

For 8-oxoGuo, fiberglass workers, both Italian and Saudi Arabian gasoline workers and Painters after the working shift (four groups).

These results seem to suggest that 8-oxoGuo could be the most sensitive biomarker to the short term, reversible effects of exposure to chemical and physical agents even in conditions that could be considered safe. The fact that RNA is single stranded could support the hypothesis that in this molecule the guanine is more exposed to oxidation than in DNA, where the double strand sterically protects the molecules of the bases: moreover, in contrast to the active repair mechanisms existing for DNA, repair for oxidatively damaged RNA has not been found. Our findings confirm previous hypotheses that in various systems the levels of oxidatively generated damage to RNA is higher than that to DNA of the same source [12].

## 5. Conclusions

Many studies in the field of occupational health consider the three biomarkers here studied, without further exploring the differences among them. Other studies reduce their analyses to the two nucleosides, (8-oxodGuo and 8-oxoGuo) as 8-oxoGua is much more variable and less correlated to other exposure parameters. Some studies consider only 8-oxodGuo, which is by far the most studied, especially in clinical studies, and also more often shows correlation with cancer biomarkers or other endpoints.

The objective of this study was to compare the levels of the considered three biomarkers in the urine of different groups of workers, and with a general population group, and to propose an explanation for the different abundance of each biomarker among the groups.

Data indicate that both DNA and RNA oxidation are produced in occupational settings, but with a slightly different behavior.

Oxidized deoxy-guanosine from DNA comes from the sum of two different mechanisms, repair and turnover (where repair seems to be prevalent), and is useful as exposure/effect biomarker for chronic occupational exposures like to benzene and styrene exposure of gasoline pump attendants and fiberglass workers respectively.

Oxidized guanosine from RNA comes only from turnover, and this could make it more suitable to study the difference between before and after working oxidation levels, and maybe, just because its turnover is quicker, it seems to be most sensitive to short-term exposure variation.

Concerning 8-oxoGua, which is more variable and more complex to understand, as it can be formed both from DNA and from RNA turnover, we still do not find a convincing interpretation of our results.

According to our knowledge, this is the first study that compares the three biomarkers of nucleic acid oxidation, in the urine of more than two different categories of workers, as most studies consider only one occupational activity or one causating agent.

A limitation of the study is the different numerosity of the groups: this a common problem in occupational exposure studies, where the researchers cannot choose the number of subjects, and is often due to the real size of the workforce.

This study is the comparison of data partly from published papers and partly from new experiments. All this data have been obtained in the same laboratory, using the same sampling and analysis methods and the same instrument, so we did not consider is to be a meta-analysis but rather an actual experimental study.

Results of this work confirm that 8-oxodGuo is a valuable biomarker for assessing occupational exposure to dangerous chemicals. Occupational exposure to chemical agents and hyperbaric atmospheres produces a measurable level of oxidatively generated damage to DNA and RNA, which is repairable in the case of DNA. Even in conditions which are regarded as not dangerous there is a detectable increase in the biomarkers concentration after a working shift, although this is still within the range measured in the general population. The urinary 8-oxoGuo, that is related to RNA oxidation, seems to be the most suitable biomarker in order to detect these short-term, reversible effects.

Further research needs are the detection of these biomarkers at different time points to know more about their excretion kinetics, and in the same individuals when they are at rest (not working). An interesting application will be to study the possible effect of physical agents such as high and low temperatures, vibrations and exposure to electromagnetic fields on these biomarkers.

## Figures and Tables

**Table 1:** Characteristic of the study groups.

Group	Subject	n.	Smokers (%)	Age (Years)	Males	Females	Urine Sampling
**1**	**Gasoline pump attendants (Saudi Arabia)**	29	7 (24.14)	20–45	29	0	After work-shift
**2**	**Gasoline pump attendants (Italy)**	102	44 (43.14)	22–63	69	33	After work-shift
**3**	**Fiberglass reinforced plastic workers (Italy)**	24	8 (33.33)	30–50	6	18	After work-shift
**4**	**Ship Painters (Bangladesh)**	17	5 (29.41)	21–54	17	0	Before and after work-shift
**5**	**Divers (Italy)**	6	2 (33.33)	42–63	5	1	Before and after diving
**6**	**Rome researchers (Italy)**	2	0 (00.00)	20–40	1	1	Over one month
**7**	**General population (Italy)**	132	24 (18.18)	35–69	68	64	Spot sampling

**Table 2:** Inter and intra individual variability for the concentrations of the oxidation biomarkers.

Parameter	Males		Females	
	Inter-Individual Variability	Intra-Individual Variability	Inter-Individual Variability	Intra-Individual Variability
**n. subjects**	68	1	64	1
**n. urine samples/subject**	1	22	1	16
**8-oxoGua**				
**% CV**	96.0	81.4	94.0	74.1
**8-oxoGuo**				
**% CV**	40.5	99.0	49.0	121.1
**8-oxodGuo**				
**%CV**	44.2	51.0	42.0	157.0

**Table 3:** Concentrations (µg/g creatinine) of the oxidation biomarkers in all groups.

Group Number	1	2	3	4		5		6	7
Group Name	Gasoline (Saudi Arabia)	Gasoline (Italy)	Fiberglass Workers (Italy)	Painters (Bangladesh)		Hyperbaric Atmosphere (Italy)		CARE Researchers Rome (Italy)	General Population (Italy)
				***Before Work***	***After Work***	***Before Diving***	***After Diving***		
**8-oxoGua *(µg/g Creatinine)***									
**Mean (SD)**	55.92 (63.61)	81.83 (60.99)	100.89 (78.82)	13.83 (15.41)	11.20 (13.69)	25.60 (14.06)	27.18 (4.78)	85.61 (73.28)	36.29 (35.81)
**5th**	1.92	17.05	14.75	0.33	0.01	11.73	23.43	17.64	6.13
**50th**	25.45	61.75	81.10	9.66	6.20	22.39	25.46	60.30	25.87
**95th**	172.97	207.49	268.54	34.04	32.53	45.55	34.17	216.40	105.20
**8-oxoGuo (*µg/g Creatinine)***									
**Mean (SD)**	29.16 (15.70)	10.63 (5.54)	34.00 (9.95)	10.27 (6.87)	16.13 (6.12)	21.45 (6.59)	36.18 (8.64)	1.95 (2.15)	12.19 (5.56)
**5th**	8.27	4.09	22.48	5.03	10.04	13.66	27.88	0.25	4.92
**50th**	26.97	9.00	31.14	8.13	14.72	22.74	34.90	1.42	11.59
**95th**	55.98	19.85	50.24	21.13	28.31	28.78	48.27	7.16	21.27
**8-oxodGuo (*µg/g Creatinine)***									
**Mean (SD)**	15.15 (9.10)	4.07 (1.69)	27.80 (11.82)	2.86 (0.89)	5.53 (1.90)	3.01 (0.78)	5.97 (1.20)	5.85 (10.40)	7.83 (3.47)
**5th**	5.87	1.66	14.74	1.67	3.28	1.98	4.92	0.89	3.52
**50th**	13.72	3.8	23.72	2.78	5.59	3.18	5.61	3.25	7.26
**95th**	27.22	7.05	50.04	4.25	8.17	3.86	7.58	14.97	14.92

**Table 4:** ANOVA results for the three oxidative stress biomarkers.

**Variable 8-oxoGua**	**Sum of Squares**	**df**	**Mean Square**	**F**	**Sign.**
**Group**	121,538.1	4	30,384.52	13.608	0
**Sex**	16,234.35	1	16,234.35	7.271	0.007
**Smoking**	34,922	2	17,461	7.82	0
**Ethnicity**	50.08	1	50.08	0.022	0.881
**Age**	3993.45	1	3993.45	1.788	0.182
**Variable 8-oxoGuo**	**Sum of Squares**	**df**	**Mean Square**	**F**	**Sign.**
**Group**	10,575.47	4	2643.868	71.581	0
**Sex**	8.411	1	8.411	0.228	0.634
**Smoking**	68.656	2	34.328	0.929	0.396
**Ethnicity**	35.843	1	35.843	0.97	0.325
**Age**	475.619	1	475.619	12.877	0
**Variable 8-oxodGuo**	**Sum of Squares**	**df**	**Mean Square**	**F**	**Sign.**
**Group**	9678.911	4	2419.728	136.495	0
**Sex**	121.486	1	121.486	6.853	0.009
**Smoking**	8.059	2	4.029	0.227	0.797
**Ethnicity**	1.57	1	1.57	0.089	0.766
**Age**	5.38	1	5.38	0.304	0.582

**Table 5:** Linear regression model results for the three oxidative stress biomarkers.

**Variable Log(8-oxoGua)**	**β Coeff**	**Standard Error**	***t* Value**	**Pr(>|t|)**
**Group 1 (Gasol SA)**	−0.02865	0.23744	−0.121	0.904
**Group 2 (Gasol Ita)**	0.91558	0.15263	5.999	5.46 × 10^−9^ ***
**Group 3 (Fiberglass)**	1.03290	0.25692	4.020	7.28 × 10^−5^ ***
**Group 4 (Painters before)**	−1.42340	0.29834	−4.771	2.81 × 10^−6^ ***
**Group 4 (Painters end)**	−2.37919	0.29834	−7.975	2.85 × 10^−14^ ***
Multiple R-squared: 0.3557, Adjusted R-squared: 0.3455, F-statistic: 34.79 on 5 and 315 DF, *p*-value: <2.2 × 10^−16^				
**Variable Log(8-oxoGuo)**	**β coeff**	**Standard Error**	***t* Value**	**Pr(>|t|)**
**Group 1 (Gasol SA)**	1.36063	0.13277	10.248	<2 × 10^−16^ ***
**Group 2 (Gasol Ita)**	0.40612	0.08595	4.725	3.50 × 10^−6^ ***
**Group 3 (Fiberglass)**	1.66304	0.14356	11.584	<2 × 10^−16^ ***
**Group 4 (Painters before)**	0.33755	0.16652	2.027	0.0435 *
**Group 4 (Painters end)**	0.89502	0.16652	5.375	1.52 × 10^−7^ ***
Multiple R-squared: 0.409, Adjusted R-squared: 0.3994, F-statistic: 42.63 on 5 and 308 DF, *p*-value: <2.2 × 10^−16^				
**Variable Log(8-oxodGuo)**	**β Coeff**	**Standard Error**	***t* Value**	**Pr(>|t|)**
**Group 1 (Gasol SA)**	0.61414	0.08827	6.957	2.02 × 10^−11^ ***
**Group 2 (Gasol Ita)**	−0.65439	0.05674	−11.532	<2 × 10^−16^ ***
**Group 3 (Fiberglass)**	1.27724	0.09552	13.372	<2 × 10^−16^ ***
**Group 4 (Painters before)**	−0.96230	0.11091	−8.676	2.23 × 10^−16^ ***
**Group 4 (Painters end)**	−0.30857	0.11091	−2.782	1.52 × 10^−7^ ***
Multiple R-squared: 0.6453, Adjusted R-squared: 0.6397, F-statistic: 114.6 on 5 and 315 DF, *p*-value: <2.2 × 10^−16^				

* statistically significant; *** highly statistically significant.
